# Microbiology of cardiac implantable electronic device infections in Calgary, Canada

**DOI:** 10.1017/ash.2024.9

**Published:** 2024-01-25

**Authors:** Teagan King, Derek S. Chew, Jenine Leal, Kristine Cannon, Zuying Zhang, Elissa Rennert-May

**Affiliations:** 1 Department of Medicine, University of Calgary, Calgary, AB, Canada; 2 Department of Community Health Sciences, University of Calgary, Calgary, AB, Canada; 3 O’Brien Institute for Public Health, University of Calgary, Calgary, AB, Canada; 4 Libin Cardiovascular Institute, University of Calgary, Calgary, AB, Canada; 5 Department of Cardiac Sciences, University of Calgary, Calgary, AB, Canada; 6 Department of Microbiology, Immunology and Infectious Diseases, University of Calgary, Calgary, AB, Canada; 7 Infection Prevention and Control, Alberta Health Services, Calgary, AB, Canada; 8 Snyder Institute for Chronic Diseases, University of Calgary, Calgary, AB, Canada

## Abstract

The microbiology of cardiac implantable electronic device (CIED) infections in Calgary, Alberta was described, identifying 50 infections from 2013 to 2019. The majority were *Staphylococcus aureus* (40.0%). There is significant economic burden, mostly related to inpatient costs, associated with CIED infections. However, there were no significant differences in costs stratified by organism.

## Introduction

Complex cardiac implantable electronic device (CIED) infections are a serious complication associated with morbidity, mortality, and increased healthcare costs. Complex infections are defined as deep incisional surgical site infections (SSI) of the fascia or muscle (excluding skin), and organ, or deep space SSIs.^1^ This would include pocket infections, valvular/lead endocarditis +/− bacteremia, and isolated bacteremia as long as standardized criteria are met.^1^ The epidemiology of CIED infections in Alberta, Canada, was recently described.^2^ The objective of this study was to describe the microbiology of CIED infections in the Calgary region and to understand health resource use and costs by causative organism.

## Methods

### Study overview

This retrospective cohort study included adult patients (ie, age ≥18 years) who underwent CIED implantation, including pacemakers (PM), implantable cardiac defibrillators (ICD), or cardiac resynchronization therapy (CRT), in Calgary, Alberta between January 1, 2013, and December 31, 2019 and who subsequently developed a complex SSI.^1^ Patients who met inclusion criteria were followed with outcome ascertainment at 1 year. Ethics approval for this study was obtained from the University of Calgary Health Research Ethics Board (REB20-2186).

### Data sources

All CIED implantations conducted within Calgary, Alberta are followed by Alberta Health Services’ (AHS) Infection Prevention and Control (IPC) department for routine postoperative infection surveillance. IPC surveillance data were used to identify CIED-associated complex SSIs and microbiology. Baseline cohort characteristics, such as Elixhauser comorbidities,^3^ and 1-year outcomes were determined using AHS Analytics administrative data, including the Discharge Abstract Database (inpatient hospitalizations), the National Ambulatory Care Reporting System (outpatient hospital and emergency department (ED) visits), and AHS Corporate Finance (inpatient micro-costing data). Costs for outpatients, including ED visits, day medicine, or day surgery visits, were determined by gross-costing methods, described through patient-level Resource Intensity Weights and the ‘Cost of a Standard Hospital Stay’ indicator.^4^

## Outcomes

The primary study end point was the causative organism of CIED infection. Secondary end points included cumulative healthcare costs at 1 year, frequency of hospital admissions, inpatient length of stay (LOS), and frequency of outpatient visits.

### Statistical analyses

Patient characteristics and causative microbiology were summarized using descriptive statistics, including proportions, means, and standard deviations (SD), as appropriate. Health resource use and costs were described for the cohort and stratified by causative organism. Generalized linear models (GLM) with Gamma distribution and log link were used to analyze costs. GLM with Poisson distribution and log link were used for admissions, LOS, and outpatient visits. R “margins” package calculated cost differences between groups and adjusted for variables: *S. aureus* vs. other organisms, age, sex, number of comorbidities, device type (PM, ICD, or CRT), and generator change. Costs were adjusted to 2022 Canadian dollars.

## Results

Of the 3536 patients who underwent CIED implantation and had a subsequent inpatient admission, 50 developed complex SSIs within 1 year of surgery: 42 culture-positive and 8 culture-negative.

**Figure 1.**
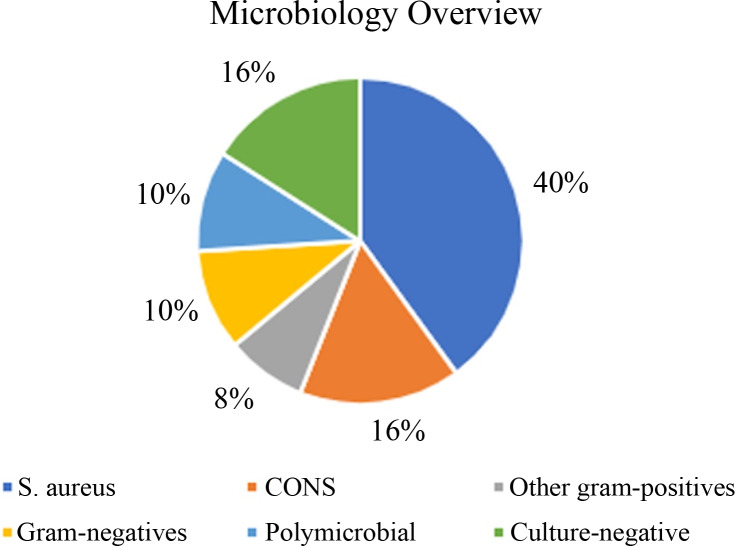
Microbiology of complex cardiac implantable electronic device infections in Calgary, Alberta.

Of the 50 SSIs, the most isolated organism was *Staphylococcus aureus (S. aureus)* from 20 patients (40.0%): 19 methicillin-sensitive and 1 methicillin-resistant (MRSA); 8 (16.0%) coagulase-negative *Staphylococcus* (CoNS); 4 (8.0%) other gram-positives; 5 (10.0%) gram-negatives; 5 (10.0%) polymicrobial, 4 of which included a *Staphylococcus* species; and 8 (16.0%) culture-negative infections (Figure 1).

Mean cumulative one year healthcare costs were highest for culture-negative infections at $109,055 (SD $84,362), compared to *S. aureus* infections at $83,996 (SD $47,444) and CoNS at $67,802 (SD $22,121). The mean number of hospital admissions was similar by causative organism; however, LOS differed by microbiology: 30 days in the *S. aureus* group and 52 days for other gram-positives. Results are summarized in Table 1.

**Table 1.** Costs, inpatient admissions, length of stay, and outpatient visits by causative organism in CIED infections

Combined Organisms		Total Costs ($)		Inpatient Costs ($)		Outpatient Costs ($)	
Organism	#	Mean (SD)	Median (IQR)	Mean (SD)	Median (IQR)	Mean (SD)	Median (IQR)
*S. aureus*	20	83,996 (47,444)	76,671 (87,675)	63,579 (45,088)	54,072 (47,000)	20,416 (17,151)	13,471 (20,683)
CoNS	8	67,802 (22,121)	68,768 (32,009)	53,462 (17,024)	49,872 (28,946)	14,341 (9,701)	11,776 (5,588)
Polymicrobial	5	95,682 (63,309)	80,226 (85,549)	83,034 (60,533)	67,550 (72,397)	12,648 (6,886)	14,137 (5,042)
Gram-negative	5	73,528 (29,886)	83,411 (55,768)	65,668 (31,445)	75,466 (47,988)	7,860 (5,842)	7,946 (5,924)
Other gram-positives	4	106,234 (120,609)	53,746 (72,183)	98,484 (122,226)	47,951 (81,791)	7,750 (5,618)	8,619 (6,784)
Culture-negative	8	109,055 (84,362)	83,809 (81,951)	83,319 (65,324)	70,984 (81,249)	25,735 (28,360)	25,114 (22,845)

		Inpatient Admissions (#)		LOS (days)		Outpatient Visits (#)	
Organism	#	Mean (SD)	Median (IQR)	Mean (SD)	Median (IQR)	Mean (SD)	Median (IQR)
*S. aureus*	20	3 (2)	2 (1)	30 (29)	20 (18)	20 (14)	18 (16)
CoNS	8	3 (2)	3 (1)	27 (8)	28 (9)	29 (28)	16 (24)
Polymicrobial	5	3 (2)	2 (4)	44 (45)	27 (26)	17 (12)	18 (7)
Gram-negative	5	2 (1)	2 (1)	25 (20)	22 (12)	18 (13)	20 (16)
Other gram-positives	4	3 (3)	2 (2)	52 (76)	16 (44)	18 (16)	18 (24)
Culture-negative	8	2 (2)	2 (0)	41 (38)	24 (27)	41 (43)	20 (60)

Note. CoNS, coagulase-negative *Staphylococcus*; *S. aureus, Staphylococcus aureus*.

In the multivariable analyses, predictors of greater cumulative 1-year costs included increasing age (OR 1.11 per 10 years, 95% CI 1.04–1.18, *p* = 0.002) and ≥6 comorbidities (OR 1.89, 95% CI 1.04–3.42, *p* = 0.036). Multimorbidity (≥6) was also associated with increased odds of hospital admission (OR 2.31, 95% CI 1.07–4.99, *p* = 0.034), longer LOS (OR 3.33, 95% CI 1.59–7.00, *p* = 0.001), and odds of outpatient visits (OR 3.85, 95% CI 2.25–6.59, *p* < 0.001). There were no significant differences in costs, admissions, LOS, or outpatient visits comparing *S. aureus* to other organisms.

## Discussion

In this study, we report the epidemiology of 50 complex CIED SSIs in patients who underwent inpatient CIED implantation in Calgary, Alberta between 2013 and 2019. *Staphylococcus* species were the most common causative organisms, identified in over two-thirds of infections. There were no fungal or mycobacterial infections. Our microbiologic profile was similar to other published CIED infection microbiology,^5–7^ where *Staphylococcus* species predominate. MRSA infections in our cohort were significantly lower than reports from the United States,^6,7^ reflective of the lower burden of MRSA in Canadian hospitals.^8^

Despite the aggressive clinical course of *S. aureus* infections, complex CIED SSIs due to *S. aureus* were not associated with the highest 1-year overall costs (ie, mean total cost $83,996 for *S. aureus* compared to $95,682 for polymicrobial infections and $109,055 for culture-negative infections). Although the costs associated with CIED infections by microbiology is not well described, our results differ from other literature reporting higher costs associated with *S. aureus* SSI in other body sites.^9^ Further, although cost differences between *S. aureus* and other organisms were not significant, ≥6 comorbidities were associated with higher total costs and longer LOS. It may be that the polymicrobial and culture-negative infection cohorts were more comorbid, with complicated hospital courses leading to higher overall costs. Overall, the LOS associated with these infections was quite prolonged (Table 1). Patients do have access to an outpatient intravenous antibiotic therapy program to help discharge them prior to completion of antibiotic courses, and more work should be done to explore the reasons for this extended LOS and ways to streamline discharges. These secondary outcomes need to be interpreted cautiously as the numbers of patients in these groups was small.

Our study’s strengths included using gold-standard IPC surveillance data to identify infections. This adds to the small body of Canadian literature regarding the microbiology of CIED infections.^10^ Gold-standard micro-costing data for inpatient costs were used where possible, in addition to gross costing, to create the most accurate representation of healthcare costs. Our study was limited by the small number of infections and subsequent microbiologic results available, limiting interpretation of differences in outcomes between organisms. Our study also did not explore management of these infections, including antibiotic choices and duration and CIED removal. Although typically CIEDs would be removed for these complex infections, this should be assessed in future studies as well as the timing to device removal and reimplantation.

The microbiology of CIED infections in Calgary, Alberta is largely driven by *Staphylococcus* species, similar to published reports. There is a significant economic burden, mostly related to inpatient costs, associated with complex CIED SSIs; however, *S. aureus* did not lead to the greatest direct health costs at 1-year compared to other organisms.

## Data Availability

The data used in this work is the property of Alberta Health Service and cannot be publicly accessed. With appropriate ethics approval and data disclosure agreements with Alberta Health Services, the authors can release data upon request.
